# Efficacy of a small-volume blood culture diversion device across three wards: a 6-month retrospective review

**DOI:** 10.1128/spectrum.00593-26

**Published:** 2026-06-09

**Authors:** Hallie H. Dolin, Alyssa M. Krupp, Amrita R. John, Sree S. Cherian, Gina R. Lewin, Elie A. Saade, Eric M. Ransom

**Affiliations:** 1 Department of Pathology, University Hospitals Cleveland Health, Cleveland, Ohio, USA; 2 Department of Internal Medicine, University Hospitals Cleveland Health, Cleveland, Ohio, USA; 3 Department of Pathology, Case Western Reserve University School of Medicine, Cleveland, Ohio, USA; 4 Center for Global Health and Diseases, Case Western Reserve University School of Medicine, Cleveland, Ohio, USA; 5 Case Western Reserve University-Cleveland VA Medical Center for Antimicrobial Resistance and Epidemiology, Cleveland, Ohio, USA; University of Kentucky, Lexington, Kentucky, USA

**Keywords:** blood culture, contamination, blood culture diversion device, initial specimen diversion device, Kurin, NHSN

## Abstract

Blood culture contamination (BCC) is a challenging issue that can negatively impact patients and healthcare costs. The Clinical and Laboratory Standards Institute (CLSI) historically encouraged a BCC rate of <3%, although recent evidence suggests <1% is achievable. One approach to reducing BCC is initial specimen blood diversion devices (ISDDs). ISDDs prevent the initial blood, which may contain contaminating microorganisms, from inoculating the blood bottles. The ISDD literature remains limited, particularly for evaluating performance across different wards. Here, the Kurin Lock ISDD was evaluated in an emergency department (ED), cardiothoracic intensive care unit (CTICU), and medical intensive care unit (MICU). A comparison of 6 months pre- and post-implementation revealed a 35.3% BCC reduction overall (*P* = 0.016), with variation across wards: ED 41.0% (*P* = 0.043), CTICU 38.7% (*P* = 0.20), and MICU 20.3% (*P* = 0.61). Device compliance rates varied by ward: ED (62.5%), CTICU (32.2%), and MICU (35.4%). When post-implementation cultures were segregated by ISDD usage, the MICU BCC rate was 0.39% versus 3.02% without ISDD usage (*P* = 0.025). The ED and MICU also had multiple months below 1%. The CTICU did not have a month below 1% nor a significant BCC rate difference post-implementation with or without ISDD usage (2.52% and 3.19%, *P* > 0.99). Furthermore, an estimated $170,378 of contamination costs were avoided using a cost per contamination of $6,553 from a prior meta-analysis. Given our low ISDD compliance, contamination rates and financial savings can still be improved. Taken together, ISDDs are a promising approach to lower BCC rates and costs, although impact may vary by ward.

**IMPORTANCE:**

Healthcare systems continue to strive toward a <1% blood culture contamination rate. Initial specimen blood diversion devices (ISDDs) are a promising approach to achieve this goal; however, data remain limited on small-volume ISDDs, especially in different hospital ward settings. Here, we found the small-volume Kurin Lock ISDD reduced blood culture contamination rates and theoretically avoided contamination costs, despite low ISDD compliance. Importantly, performance varied considerably across an emergency department and two intensive care units. While the presented findings support ISDD usage, health systems must be mindful of variable ward performance and manage expectations accordingly.

## INTRODUCTION

Blood culture contamination (BCC) is a significant issue in healthcare worldwide. An estimated 20%–50% of all positive blood cultures are contaminated (1–4). The negative consequences of BCC for patients and healthcare systems can be significant. For example, contaminated cultures can lead to extended hospital stays (1), and patients with presumed systemic infections are likely to be treated with empiric antimicrobial agents, such as vancomycin, with potential severe side effects requiring therapeutic monitoring (1, 5, 6). BCC also carries a substantial financial burden, including unnecessary diagnostic testing, treatments, and general patient care (1, 5, 7). While financial estimates vary in the literature, increased costs per contamination are consistently estimated to be in the thousands of dollars, potentially reaching $10,000 or more per culture, and averaging approximately $6,553 based on the most comprehensive recent meta-analysis (1, 5, 8).

BCC is largely attributed to the function of the blood draw collection process. BCC is most frequently from commensal skin flora, such as staphylococci, streptococci, and micrococci (1, 2, 9). Even with proper skin preparation and collection techniques, contamination can still occur. Initial specimen diversion devices (ISDDs) have shown to be effective at reducing BCC rates by sequestering a small amount of blood at the beginning of each draw (4, 10–14). The underlying principle is that the entry of a hollow needle into the vasculature can create a skin plug with flora located in the inner dermis layer, which may not have succumbed to the antiseptic agent.

The Kurin Lock device, which sequesters the first 0.15 mL of blood per draw, has been found in ED studies to reduce BCC significantly (11, 14, 15). Additional peer-reviewed literature is needed to evaluate the performance of this small-volume ISDD in non-ED settings and evaluate the financial considerations. This study evaluated the Kurin ISDD for 6 months in three wards simultaneously within a hospital system, all with different patient populations, staffing, and blood draw workflows. In addition, this study investigated theoretical financial savings when using the ISDD to avoid costly blood culture contamination.

## MATERIALS AND METHODS

### Study design

The University Hospitals Cleveland Medical Center institutional review board (IRB) approved this study and granted a waiver of consent. To gain insight into ISDD impact across different patient populations, staffing, and blood draw workflows, three wards were selected for the intervention within the University Hospitals Cleveland Health system: a community hospital emergency department (ED), an academic center cardiothoracic intensive care unit (CTICU), and an academic center medical intensive care unit (MICU). Blood culture results and patient information were obtained from the electronic medical record (EMR) during the pre-intervention (January 2023–June 2023) and intervention (August 2023–January 2024). The training month of July 2023 was excluded.

### Blood culture collection and testing

Blood cultures were drawn per routine standard-of-care practices. All patients were over the age of 18 years, and every blood culture set had an aerobic and anaerobic bottle. Staff were trained to use the Kurin Lock ISDD (Kurin Inc., San Diego, CA, USA) in July 2023 by device representatives, followed by 6 months of unsupervised use and limited targeted re-education. In all three wards, phlebotomy or nursing can draw the blood cultures; however, the majority were drawn by nursing in the CTICU and MICU and by phlebotomy in the ED. Compliance reminders with usage and contamination events were sent monthly to ward managers. To monitor ISDD compliance, each device was pre-packaged from the manufacturer with all necessary components, eliminating the need to combine sources of supplies. Each kit (21-gauge M-22121 peripheral, 23-gauge M-22123 peripheral, and M-PIV18 for initial intravenous placement) contained record-keeping packaging detailing the device number and type. The blood collector used the record-keeping packaging to record collector’s name and initials/signature (also confirmed in EMR), the time and date of blood collection, patient identifiers, and specimen ID. Device compliance was determined by lab receipt of the record-keeping packaging.

Blood cultures were collected and tested following routine standard-of-care practices. Briefly, blood cultures were drawn into aerobic (bottle types: SA or FA plus) and anaerobic (bottle types: SN or FN plus) blood culture collection bottles (BacT/ALERT, bioMérieux, Marcy-l'Étoile, France) and incubated in the BacT/ALERT VIRTUO automated growth detection system (bioMérieux, Marcy-l'Étoile, France). Positive blood cultures underwent Gram staining and were plated onto sheep’s blood, chocolate, MacConkey, and CDC anaerobic agar plates. Aerobic agars were incubated in the BD Kiestra Total Lab Automation System (BD Biosciences, Franklin Lakes, NJ, USA). Anaerobic agars were incubated using the BD GasPak EZ anaerobe pouch system with an indicator. Microbial identification was performed with the MALDI-TOF MS Biotyper (Bruker Scientific, Billerica, MA, USA).

### Contamination definitions and calculations

Cultures were categorized as contaminated or non-contaminated based on National Healthcare Safety Network (NHSN) criteria, available at the Centers for Disease Control and Prevention website (16). Briefly, a culture was deemed contaminated if an organism from the NHSN 2024 common commensal list (contaminant list) was found in only one of two (or more) cultures collected within 24 h. If an organism not on the list was present in the culture (“pathogen”), the culture definition did not change. Cultures were considered non-contaminated if the common contaminant was recovered in multiple blood culture sets collected within 24 h. Each blood culture set included one aerobic and one anaerobic bottle. Any cultures that did not have a companion culture collected within 24 h were excluded from analysis.

Both pre-intervention and intervention contamination rates were calculated by dividing the contamination counts by the total number of cultures. For data collected during the intervention, usage of the ISDD, or lack thereof, was noted for each culture. Compliance rates were calculated by dividing the number of cultures drawn with the ISDD by the total number of cultures.

### Chart review and patient information

Patients were categorized as “with contaminants” (C) if a blood culture had a common commensal and “without contaminants” (NC) if they had none. The definition of contamination was the same as stated above: if a commensal organism was found in only one of two or more cultures collected within 24 h, the culture was deemed contaminated. If a patient’s results appeared during multiple months and/or in multiple wards, the patient was only noted in the first month and/or ward of appearance. All patient charts were reviewed, and demographic data were collected. Chosen metrics included sex, race, obesity, cancer (current or past), coronary artery disease, chronic kidney disease, chronic obstructive pulmonary disease, congestive heart failure, diabetes, liver disease, receipt of antimicrobial agent(s), receipt specifically of vancomycin as a sub-analysis, and whether the patient died during their stay.

### Cost calculations

To estimate the contamination count assuming no intervention, the average pre-intervention contamination rate was multiplied by the number of total intervention cultures. The resulting estimated contamination count was then compared with the actual number of contaminated cultures during the intervention. The estimated difference in cost (EDC) was calculated using $6,553 per contaminated culture, which was calculated by averaging the reported costs per contaminated culture in a large comprehensive meta-analysis study from 2009 (1). The range was $4,385–8,720 (1). To adjust for inflation, the cost per contaminated culture was updated using the CoinNews Media Group inflation calculator at usinflationcalculator.com. The EDC 100% Compliance adjusts the evaluation to assume 100% compliance.

### Statistical analysis

Statistical analysis was performed with GraphPad Prism 10 using Fisher’s exact test.

## RESULTS

### Pre-Intervention blood culture positivity and contamination rates

Hospital wards can be strikingly different environments with variable blood culture workflows and contamination rates. Here, the small-volume Kurin Lock ISDD was implemented in an ED, CTICU, and MICU. The contamination rate pre-intervention across all three wards was 3.62% (83 of 2,295 cultures). The ED had a pre-intervention contamination rate of 3.82% (44 of 1,151 cultures). The CTICU had a pre-intervention contamination rate of 4.85% (19 of 392 cultures). The MICU had a pre-intervention contamination rate of 2.62% (20 of 762 cultures). In brief, these are three wards with notably different contamination rates, which are likely to reflect different blood culture collection practices, workflows, and collector expertise.

### Contamination rates during intervention

**Fig 1:**
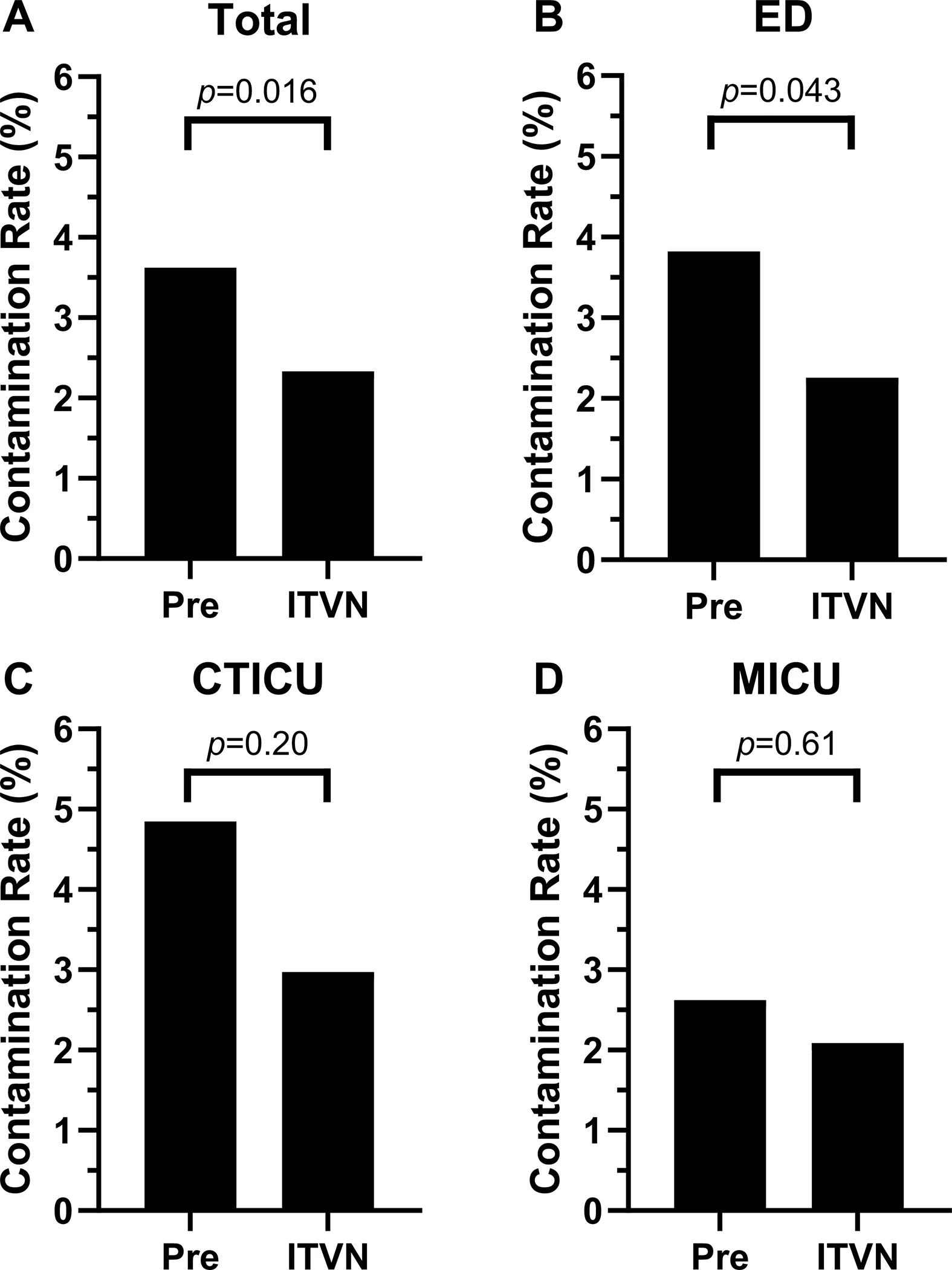
BCC rates pre-intervention (Pre) and intervention (ITVN) in total (**A**), ED (**B**), CTICU (**C**), and MICU (**D**).

After a 1-month ISDD training period, the following 6 months were considered the intervention period to evaluate performance. The contamination rate across all wards decreased by 35.3% from 3.62% (83 of 2,295 cultures) to 2.33% (47 of 2,018 cultures) (Fig. 1A). The ED had a 41.0% reduction from 3.82% (44 of 1,151 cultures) to 2.26% (21 of 931 cultures) (Fig. 1B). The monthly minimum and maximum contamination rates for the ED were 0.85% (November) and 5.0% (September), respectively. The CTICU had a 38.7% reduction from 4.85% (19 of 392 cultures) to 2.97% (11 of 370 cultures) (Fig. 1C). The monthly minimum and maximum contamination rates for the CTICU were 1.70% (August) and 5.00% (December), respectively. The MICU had a 20.3% reduction from 2.62% (20 of 762 cultures) to 2.09% (15 of 717 cultures) (Fig. 1D). The monthly minimum and maximum contamination rates for the MICU were 0.00% (January) and 5.7% (September), respectively. While statistical significance was not demonstrated in the CTICU (*P* = 0.20) and MICU (*P* = 0.61) during the intervention, the ED and combined three-ward total had *P* values of 0.043 and 0.016, respectively.

### Compliance monitoring

A possible explanation for the lack of statistical significance above is low ISDD compliance. This was supported by reports from study observers and ISDD inventory tracking. To monitor ISDD compliance, blood culture collectors were trained and reminded to send the device packaging with collector and patient information. Compliance rates were variable over the course of the study and were inconsistent from month to month, both within and between wards (Table 1).

**TABLE 1:** Monthly ISDD compliance rates by ward

Ward	ISDD compliance rates by intervention month						Overall
	1	2	3	4	5	6	
ED	55.6% (89/160)	48.5% (94/194)	40.2% (45/112)	72.9% (86/118)	80.4% (135/168)	74.3% (133/179)	62.5% (582/931)
CTICU	51.7% (30/58)	23.2% (13/56)	22.2% (18/81)	24.3% (17/70)	52.0% (26/50)	27.3% (15/55)	32.2% (119/370)
MICU	32.8% (39/119)	25.5% (27/106)	23.0% (26/113)	35.2% (45/128)	53.2% (66/124)	40.2% (51/127)	35.4% (254/717)

While ISDD compliance was lower than the target of 80%, it provided an opportunity for a sub-analysis: to assess whether statistical significance differed during the intervention when divided into categories of “with ISDD” (*n* = 955) and “without ISDD” (*n* = 1,063). The mean contamination rates with all cultures from all wards were, respectively, 1.36% (*n* = 13) and 3.20% (*n* = 34) (*P* = 0.0073). For the ED, the contamination rate for “with ISDD” was 1.55% (9 of 582 cultures) and “without ISDD” was 3.44% (12 of 349 cultures), which neared statistical significance (*P* = 0.070). For the CTICU, contamination rate for “with ISDD” was 2.52% (3 of 119 cultures), and “without ISDD” was 3.19% (8 of 251 cultures), which was not statistically significant (*P* > 0.99). For the MICU, contamination rates for “with ISDD” was 0.39% (1 of 254 cultures) and “without ISDD” was 3.02% (14 of 463 cultures), which was statistically significant (*P* = 0.025).

### Intervention contamination benchmarks

In addition to lower BCC rates, it is important that ISDD usage can help health systems achieve contamination targets below the standard 3% and, ideally, 1% thresholds. To evaluate these targets in the context of ISDD use, the number of months above and below these thresholds were examined. Each ward experienced at least a 33.3% decrease in the number of months with ≥3% contamination (66.7% to 16.7% in the ED, 66.7% to 33.3% in the CTICU, and 50% to 33.3% in the MICU) (Table 2). The MICU had the most favorable number of months <1%, three total, which was never achieved pre-implementation. The CTICU was unable to achieve a single month below 1%.

**TABLE 2:** Number and percentage of months by ward with ≥3%, 1%–2.99%, and <1% BCC during the pre-intervention (Pre) and intervention (ITVN)

Ward	Period	Months (%) with a contamination rate of		
		≥3%	1%–2.99%	<1%
ED	Pre	4 (66.7%)	1 (16.7%)	2 (33.3%)
	ITVN	1 (16.7%)	3 (50.0%)	2 (33.3%)
CTICU	Pre	4 (66.7%)	0 (0.00%)	2 (33.3%)
	ITVN	2 (33.3%)	4 (66.7%)	0 (0.00%)
MICU	Pre	3 (50.0%)	3 (50.0%)	0 (0.00%)
	ITVN	2 (33.3%)	1 (16.7%)	3 (50.0%)

To ensure that the difference between pre-intervention and intervention contamination rates was not simply the result of better surveillance, self-observation, and/or sterilization techniques after introduction of the device, contamination rates were compared between pre-intervention rates as a whole and the intervention rates without ISDD (3.62% and 3.20%, respectively). This was found to be statistically insignificant (*P* = 0.61).

### ISDD impact on positivity rates

A potential concern of ISDD usage is reduced pathogen recovery and therefore reduced overall blood culture positivity rates. Positivity rates from all positive blood cultures were compared between pre-intervention and intervention months and revealed rates actually increased to 10.46% from 7.32% pre-intervention (*P* = 0.0003).

### Isolate categorization

The number and type of isolates in positive cultures differed markedly between contaminated and non-contaminated cultures (*n* = 59 contaminated, *n* = 178 non-contaminated), as expected based on the contamination criteria. The majority of contaminated cultures contained CoNS at 79.7%, followed by *Micrococcus* spp. and *Enterococcus faecium* at 3.4% each. Additional isolates included viridans group streptococci, *Cutibacterium* spp.*, Bacillus* spp., and other coryneform bacteria (Table 3). Isolates from non-contaminated blood cultures varied more widely. *Staphylococcus aureus* composed 26.4% of total isolates. The next most common microorganisms were *Escherichia coli* at 15.2% and CoNS species at 9.0%. *Candida albicans, Klebsiella pneumoniae,* and *Enterococcus faecalis* composed 7.3%, 6.7%, and 3.9% of total isolates, respectively. The remaining microorganisms included other *Candida*, *Providencia, Proteus,* and rarer microbes, such as *Bifidobacterium* (Table 3). Total CoNS isolates were additionally identified and quantified by species (*n* = 16). The majority were *Staphylococcus epidermidis* (*n* = 10), followed by *Staphylococcus hominis* (*n* = 2).

**TABLE 3:** Representation of all unique microorganisms identified in positive cultures within the contaminated and non-contaminated groups during the intervention^*a*^

Organism	Non-contaminated culture		Contaminated culture	
	*n*	%	*n*	%
*Staphylococcus aureus*	47	26.4	1	1.7
*Escherichia coli*	27	15.2	0	0
Other coagulase-negative staphylococci	16	9.0	47	79.7
*Candida albicans*	13	7.3	0	0
*Klebsiella pneumoniae/variicola*	12	6.7	0	0
*Enterococcus faecalis*	7	3.9	0	0
*Candida parapsilosis*	5	2.8	0	0
*Proteus mirabilis*	5	2.8	0	0
*Haemophilus influenzae/parainfluenzae*	4	2.3	0	0
*Serratia marcescens* group	4	2.3	0	0
*Enterococcus faecium*	3	1.7	2	3.4
*Providencia stuartii*	3	1.7	0	0
*Pseudomonas aeruginosa*	3	1.7	0	0
*Streptococcus dysgalactiae/canis*	3	1.7	0	0
*Bacillus* spp.	2	1.1	1	1.7
*Candida* (*Meyerozyma*) *guilliermondii*	2	1.1	0	0
*Citrobacter freundii* complex	2	1.1	0	0
*Enterobacter cloacae* complex	2	1.1	0	0
Other viridans group streptococci	2	1.1	1	1.7
*Staphylococcus lugdunensis*	2	1.1	0	0
*Streptococcus agalactiae* (Group B)	2	1.1	0	0
*Streptococcus anginosus* group	2	1.1	0	0
*Streptococcus pyogenes* (Group A)	2	1.1	0	0
*Bacteroides thetaiotaomicron* group	1	0.6	0	0
*Bifidobacterium* spp.	1	0.6	0	0
*Candida tropicalis*	1	0.6	0	0
*Dialister* spp.	1	0.6	0	0
*Klebsiella* (*Enterobacter*) *aerogenes*	1	0.6	0	0
*Prevotella buccae*	1	0.6	0	0
*Sphingomonas* spp.	1	0.6	0	0
*Stenotrophomonas maltophilia* group	1	0.6	1	1.7
*Acinetobacter lwoffii*	0	0	1	1.7
*Cutibacterium* spp.	0	0	1	1.7
*Micrococcus* spp.	0	0	2	3.4
Other coryneform bacteria	0	0	1	1.7
*Rothia mucilaginosa*	0	0	1	1.7
Total	178	100	59	100

^*a*^ Organisms were classified by the definition of the cultures in which they were recovered (i.e., a pathogen and contaminant recovered from the same contaminated culture are both listed under contaminated culture).

### Patient demographics

In total, 116 unique patients with positive blood cultures were identified, 42 with at least one contaminated culture and 74 with none (Table 4). Patients with contaminated culture(s) were significantly more likely to be female (*P* = 0.035) and to have coronary artery disease (*P* = 0.033). Additionally, the increased percentage of patients with contaminated culture(s) and obesity approached significance (*P* = 0.082). There were no significant differences between patient groups in race, antimicrobial use, vancomycin use, and rates of malignancy, diabetes, CKD, CHF, COPD, or death during hospital stay.

**TABLE 4:** Select demographics/conditions for all unique patients with at least one positive blood culture set^*a*^

Demographic/condition	No contamination, % (*n*)	≥1 contaminated, % (*n*)	*P* value
Sex (female)	38% (27)	57% (24)	**0.035**
White	74% (55)	83% (35)	0.36
Obese	43% (32)	62% (26)	0.082
Cancer (current only)	23% (17)	19% (8)	0.81
Cancer (current or past)	35% (26)	29% (12)	0.54
Coronary artery disease	45% (33)	67% (28)	**0.033**
Chronic kidney disease	39% (29)	43% (18)	0.70
Chronic obstructive pulmonary disease	39% (29)	45% (19)	0.56
Congestive heart failure	45% (33)	52% (22)	0.44
Diabetes	47% (35)	45% (19)	0.85
Liver disease	24% (18)	26% (11)	0.83
Received antimicrobials during stay	97% (72)	90% (38)	0.19
Received vancomycin during stay	77% (57)	79% (33)	>0.99
Expired during stay	26% (19)	38% (16)	0.21

^*a*^ There were 74 total patients under “no contamination” column and 42 total patients under “≥1 contaminated” column. Statistically significant values are formatted in bold.

### ISDD estimated savings

The pre-intervention contamination rate was 3.62% (83 of 2,295 cultures). Thus, the estimated number of contaminants during the intervention was 73 (3.62% multiplied by 2,018 intervention cultures). Only 47 cultures (2.33%) were found to be contaminated during the intervention. The theoretically avoided number of contaminated cultures was 26 based on these calculations. Savings were estimated to be $170,378 using a cost per contamination of $6,553 from a meta-analysis using data largely between 1989 and 2009 (1). The gross savings were reduced to $153,666 after accounting for device usage cost ($17.50 multiplied by 955 ISDD cultures). Note, these calculations do not account for inflation since 2009. Re-estimating the cost savings using an inflation calculator increased the savings to $202,681–$230,597. In practice, these savings could be lower for a variety of reasons, including implementation of rapid molecular diagnostics or even a small subset of contaminated cultures that may still accrue significant expenditures due to a pathogenic microorganism also being present in the culture (see Table 3).

Given the variability of cost per contamination in the peer-reviewed literature and variability of costs at different health systems, an alternative approach is to determine the breakeven point for ISDD usage. The direct ISDD supply cost over the intervention was $16,713 ($17.50 multiplied by 955 ISDD cultures) to prevent 26 contaminated cultures. Based on these calculations, it cost the system $643 in ISDD supplies per avoided contamination. An alternative approach is to calculate cost assuming 100% compliance (2,018 intervention ISDDs) plus 10% to account for training sessions and complicated draws requiring multiple ISDDs per draw. Using this approach, the actual cost would have been $38,847 or $1,494 per contamination. A cost per contamination of $1,494 is still below most estimates of BCC costs in the literature, which ranged in one meta-analysis from $4,385 to $8,720 (1). Therefore, it is likely that implementation of ISDD results in significant net cost savings to a health system.

## DISCUSSION

Reducing BCC rates remains an important goal of healthcare systems. While the literature is growing to support the use of ISDDs, studies remain limited for small-volume ISDDs (11, 14, 15). This study evaluated the Kurin Lock device, which sequesters approximately one tenth the amount of blood as other ISDDs. Our results indicate that a small-volume ISDD is a viable method of BCC reduction in both ED and non-ED wards.

While multiple studies using ISDDs have been performed within the past decade, the majority of these have taken place only in the ED. In our analysis, almost half of the pilot cultures were drawn in a community ED (931/2018 cultures, or 46.1% of total), and the ED had a 41.0% reduction from 3.82% to 2.26% post implementation. A 2021 study evaluating four EDs within a hospital system showed a similar final BCC rate, averaging 2.1% (15). Another 6-month study taking place in a suburban ED showed a 51% decrease in BCC rate with ISDD use from 2.92% to 1.42%, with compliance rates not noted (11). Finally, a 5-month oversight period with a different device demonstrated decreased BCC rates to nearly zero in a university medical center ED (13). Given the contamination challenges and prior ISDD literature in the ED, use of ISDDs is promising.

There are limited published reports of ISDD performance in a non-ED setting. This is noteworthy since we found device performance varied by ward. For example, the CTICU contamination rates were not statistically significant (*P* > 0.99) with and without ISDD, and the CTICU was unable to achieve a single month below a 1% contamination threshold. On the other hand, the MICU contamination rates with and without ISDD were statistically significant (*P* = 0.025), and the MICU had the most favorable number of months <1%, three total. The MICU and CTICU both utilize indwelling lines, but the staff in the CTICU more frequently draw from existing lines, which was confirmed by observers. It has been shown that central line draws cause significantly higher BCC rates compared with peripheral draws (17–19). A single 2023 study using a different device included 24% ICU patients, but results were not divided by location, making it difficult to compare with our inpatient results (13). Due to the performance variation observed in our study, additional evaluation of ISDDs in non-ED settings is warranted.

An important consideration for ISDD implementation is financial. Decades of tracking have shown that BCC continues to cause a significant financial burden, with thousands of dollars wasted for a single contaminated culture (1). In this manuscript, multiple financial calculations were presented, including cost savings and a breakeven cost, which is rarely reported. Adjusting for inflation, upwards of hundreds of thousands of dollars were likely saved by elimination of 26 predicted contaminated cultures. These numbers are consistent with estimated savings in single high-volume ED settings of more than $500,000 annually (20). Here, we also found an ISDD breakeven cost of $643–$1,494, which is lower than most reported costs per contaminant estimates in the literature. Importantly, the ISDD breakeven point varies by contamination rate and will decrease at institutions with higher contamination rates. Taken together, our results show a small-volume ISDD decreases BCC-induced expenditures.

Statistically significant and near-significant differences in demographics and clinical state were found between patients with and without BCC. BCC patients were more likely to have been female and have coronary artery disease; additionally, the percentage of patients with obesity approached significance. Interestingly, obesity is associated with higher rates of skin infections, potentially in tandem with chronic inflammation and the variability of microbial species at different skin depths (21, 22). Given the skin-plug mechanism of ISDDs, differences in the microbiome within the inner layers of tissue as well as sex-specific adipose tissue distribution may play a role in BCC incidence among certain patient populations. Patient age could be another contributing factor, but was not evaluated in this study

The personnel drawing blood cultures plays a pivotal role in lowering BCC rates. In this study, these individuals were primarily nurses or phlebotomists. While a variety of staff roles can draw in these three wards, the majority were drawn by nursing in the CTICU and MICU and by phlebotomy in the ED. Previous studies have shown that BCC rates typically decrease when phlebotomists draw blood cultures due to more training and experience (1, 8). Ultimately, lowering BCC rates requires buy-in from the blood culture collector. Here, we did not observe decreased compliance or increased contamination rates in the later months of the study, although utilization was far below the 80% target. Only one month from a single ward exceeded the target utilization rate. Future work aims to investigate methods to improve this rate, and ISDD use in general, across all wards. Improved compliance rates cannot be at the expense of blood volume collected. Future work may also investigate if the added device step impacted blood volume collected, even though the amount diverted is small.

In conclusion, blood culture contamination, with its well-studied consequent negative effects, is a significant and growing problem in healthcare worldwide. Decreasing BCCs is important since contamination may negatively impact patients, an institution’s finances, and antimicrobial stewardship (23). The results of this study show small-volume ISDDs effectively decrease BCC in multiple wards and have a potential net positive financial impact, even with low compliance rates. With further compliance and device experience, ISDDs are a potential solution to mitigate BCC and significantly improve patient care and financial impact within a hospital system.

## Data Availability

Any data not presented in this article are available upon request.
